# Beyond Traditional Simulation: An Exploratory Study on the Effectiveness and Acceptability of ChatGPT‑4o Advanced Voice Mode for Communication Skills Practice Among Medical Students

**DOI:** 10.7759/cureus.84381

**Published:** 2025-05-19

**Authors:** Alifya Mukadam, Shruti Suresh, Chris Jacobs

**Affiliations:** 1 Medical Education, Great Western Hospitals NHS Foundation Trust, Swindon, GBR; 2 Psychology, University of Bath, Bath, GBR; 3 Postgraduate Medical Education, Great Western Hospitals NHS Foundation Trust, Swindon, GBR

**Keywords:** artificial intelligence, chatgpt, clinical communication training, communication skills, large language models, medical education, mixed-methods research, simulated patients

## Abstract

Background

Effective communication skills are essential for quality medical practice and patient care, yet providing sufficient practice opportunities remains challenging in medical education. Traditional approaches using standardized patients (SPs) face limitations, including high costs and logistical constraints. This study investigates the potential of conversational artificial intelligence (AI) to supplement communication skills practice among medical students.

Methodology

We conducted a mixed-methods study employing a convergent parallel design with 27 medical students from three UK universities between November 2024 and March 2025. Participants engaged in communication skills roleplay sessions using freely available ChatGPT as an SP. Pre- and post-session assessments measured perceived usefulness and self-reported confidence in three communication domains: dealing with challenging patients, breaking bad news, and counselling anxious patients. The instrument was adapted from the Immersive Technology Evaluation Measure (ITEM). Quantitative data were analyzed using non-parametric tests and ordinal logistic regression, while qualitative responses underwent reflexive thematic analysis.

Results

Self-reported confidence increased significantly across all communication domains following the ChatGPT simulation. Confidence in dealing with challenging patients increased from a median of 3 to 4 (*P *< 0.001), breaking bad news from 2 to 4 (*P *< 0.001), and counselling anxious patients from 3 to 4 (*P *< 0.001). University affiliation influenced willingness to repeat the experience (odds ratio (OR) = 0.19; 95% confidence interval (CI): 0.05-0.83) and perceived effectiveness in creating realistic scenarios (OR = 0.18; 95% CI: 0.04-0.79). Qualitative analysis revealed four themes of varying experience and expectations, valuable features (particularly detailed feedback and safe practice environment), limitations (technical challenges and restricted emotional range), and AI's complementary role in education.

Conclusions

This exploratory study provides preliminary evidence that conversational AI may enhance medical students' confidence in challenging communication skills while offering structured feedback in an accessible format. While we observed significant pre-post improvements in self-reported confidence measures, these findings should be interpreted cautiously given the small sample size, absence of a control group, and reliance on subjective self-assessments. Limitations in emotional authenticity and transcription accuracy were identified, alongside notable institutional variations in perceived realism. Despite these constraints, participants valued the psychological safety and convenience afforded by AI simulation. Our findings suggest that AI simulation may have potential as a complementary tool to expand communication skills practice opportunities, though larger controlled studies are needed to establish efficacy relative to traditional approaches. Future research should incorporate objective outcome measures and investigate the transferability of skills to real clinical encounters.

## Introduction

Effective communication skills are foundational to quality medical practice and patient care. These skills have been consistently linked to improved health outcomes, increased patient satisfaction, enhanced medication adherence, and reduced malpractice claims [1]. Despite their importance, teaching and evaluating communication skills in medical education remains challenging, particularly in providing students with sufficient opportunities for practice and feedback [2].

Traditionally, medical schools have employed standardized or simulated patients (SP) to create realistic clinical scenarios for students to practice their communication skills [3]. However, the implementation of SP programs faces several challenges, including high costs, logistics of scheduling, limited availability, and variability in actor performance [4]. The COVID-19 pandemic accelerated the experimentation of virtual and technological solutions in medical education, necessitating new approaches to teaching clinical communication skills when in-person interactions were restricted [5]. This shift has prompted educators to explore digital alternatives for communication skills training, including virtual patients (VPs) and simulation technologies.

Role play has long been established as a cornerstone method for developing clinical communication skills in medical education. This pedagogical approach allows students to practice and refine their interpersonal abilities in a controlled environment before encountering real patients [6]. Typically, role-play scenarios employ either peer students, faculty members, or trained SPs to simulate clinical encounters, enabling learners to experiment with different communication strategies and receive immediate feedback on their performance. Research has demonstrated that structured role-play exercises significantly improve students' empathic responding, history-taking techniques, and patient-centered care [7]. However, traditional role-play methods face practical constraints, including scheduling difficulties, high costs of trained SPs, limited opportunities for repeated practice, and variability in scenario delivery. These limitations have prompted educators to explore supplementary approaches that might increase practice opportunities while maintaining educational value. Digital technologies, particularly those employing conversational AI, represent a potential solution that could expand access to role-play exercises while addressing some of the logistical and resource constraints inherent in traditional methods.

Conversational agentic artificial intelligence (AI) offers a promising avenue for addressing some of these challenges. Large language models (LLMs) like ChatGPT have demonstrated capabilities in generating human-like text and engaging in natural conversations [8,9]. These conversational AI systems can process and respond to natural language input, maintain context throughout extended interactions, and exhibit agentic behavior by adapting responses based on the conversational flow and SP characteristics [10,11]. The agentic nature of modern conversational AI, which can be defined as the capability to act independently, make decisions, and respond dynamically to user input, represents a significant advancement over earlier rule-based chatbots [12]. This agency allows systems like ChatGPT to simulate patient autonomy, emotional states, and decision-making in ways that might closely approximate human behavior.

Recent research has begun to explore the use of AI chatbots in healthcare education. Studies have demonstrated that AI-based simulations could provide consistent and standardized interactions for medical students [13], and VP simulations could effectively supplement traditional clinical education approaches [14]. However, qualitative research exploring students' experiences and perceptions of AI-SPs is limited [15].

This study employs a mixed-methods approach to assess the potential utility of conversational AI (ChatGPT) as an SP for practicing challenging communication skills among medical students. Distinguishing from prior studies is the use of an advanced LLM with a naturalistic voice and low response latency. The study objective was to examine students' perceptions of ChatGPT's usefulness before and after the interaction, its effect on self-reported confidence in various communication domains, and factors that may influence students' experiences with this educational tool. By integrating quantitative measures with qualitative insights, we aim to help understand the appropriate role of such technologies in communication skills education.

## Materials and methods

Study design and participants

This mixed-methods study employed a convergent parallel design, combining quantitative pre-post assessments with qualitative thematic analysis. The cohort study was conducted between November 2024 and March 2025. A total of 27 medical students on placement at the regional hospital site were recruited from three UK universities through convenience sampling via email advertisement. Exclusion criteria were limited to inability to use supplied hardware due to physical disability. Participants were predominantly in their fourth year of medical school with a median age of 23 years (range 21-26). Specifically recruited clinical years of training as participants since they have completed foundational communication skills training, but require additional practice with the more challenging scenarios addressed in this study. This educational evaluation was reviewed by the Independent Review Board (IRB) under the UK Health Research Authority (HRA) guidelines. As the study involved the evaluation of an educational technology with medical students and did not involve patients or clinical staff providing direct patient care, it was classified as an educational improvement project. All participants provided informed consent, and data were anonymized before analysis.

Intervention

The intervention followed a structured sequence: participants completed pre-session questionnaires assessing perceived usefulness of AI for communication practice and self-rated confidence in three specific communication domains. To ensure consistent delivery across participants, all ChatGPT sessions followed a standardized protocol with pre-written prompts for each clinical scenario. Students received a brief orientation to the ChatGPT model 4o voice-to-voice conversational AI as an SP. Students spoke verbally to ChatGPT with voice input enabled and synthetic voice output. Each participant engaged in a 15-minute role-play scenario with AI acting as a SP in one of three challenging communication contexts (breaking bad news, managing difficult patients, or counselling anxious patients). Immediately following the roleplay, ChatGPT provided structured feedback on the student's communication approach. Finally, participants completed post-session questionnaires reassessing perceived usefulness and confidence levels, along with additional questions evaluating their experience. Sessions utilized a standard laptop running ChatGPT in browser mode with an external microphone and speakers in private, quiet rooms. All sessions were observed by researchers who recorded field notes on interaction patterns and technical performance.

Prompts and LLM training were pretested by running multiple prompt versions until researchers felt a realistic scenario was constructed and the avatar remained in character (Appendix). Variation to LLM output was allowable to simulate a patient responding to consultation content. 

Data collection

Data collection was conducted through self-reported assessments using a 5-point Likert scale (1 = lowest, 5 = highest). Participants completed evaluations before and after the session, measuring their perceived usefulness of AI tools for communication skills practice. The assessment also measured participants' confidence levels across three distinct clinical communication scenarios: dealing with difficult patients, breaking bad news, and counselling anxious patients. These confidence measures were collected both before and after the session to evaluate changes and were based on standardized confidence questions [16]. Additionally, post-session assessments captured participants' overall experience with the intervention using an adapted Immersive Technology Evaluation Measure (ITEM), which has demonstrated high reliability and validity [17]. This comprehensive evaluation approach allowed for direct comparison of pre- and post-session perceptions and confidence levels across multiple domains of clinical communication.

A summary of the descriptive statistics for all variables is presented in Table 1.

**Table 1:** Participants' demographics. Percentages are shown in parentheses. Universities are coded (1-3) for anonymity. SD, standard deviation; IQR, interquartile range.

Characteristic	n (%)
Age (years)	
Mean (SD)	23.0 (1.2)
Median (IQR)	23 (22-23)
Range	21-26
Gender	
Female	16 (59.3)
Male	11 (40.7)
Year of study	
Year 4	20 (74.1)
Year 5	7 (25.9)
University	
University 1	15 (55.6)
University 2	6 (22.2)
University 3	6 (22.2)

Additional observations were recorded in field notes by researchers on LLM behaviors and dynamics of interactions.

Analysis

Data were analyzed using non-parametric tests due to the ordinal nature of Likert scale responses. The Shapiro-Wilk test was used to assess normality. Pre- and post-session comparisons were conducted using the Wilcoxon signed-rank test, a non-parametric paired test.

To examine whether university affiliation and other participant characteristics influenced post-session responses, ordinal logistic regression was employed. The models included university affiliation, gender, age, and year of study as predictors. The proportional odds assumption was tested using the test of parallel lines. Results were reported as adjusted odds ratios (OR) with 95% confidence intervals (CIs).

Post hoc power analysis was conducted using the observed effect sizes calculated from pre-post differences. Cohen's d values were computed for each outcome measure and adjusted using a 0.95 correction factor for non-parametric tests. Power calculations were performed assuming a two-tailed alpha of 0.05 and the sample size of paired observations. The analysis determined the achieved statistical power for detecting the observed effects and the minimum detectable effect size given the study's sample size. For analyses involving multiple comparisons, such as university-based differences in fidelity scores, the Benjamini-Hochberg procedure was applied to control the false discovery rate with an alpha level of 0.05.

Furthermore, the internal consistency of the post-session evaluation items was assessed using Cronbach's alpha coefficient to determine the reliability of our measurement of students' perceptions of the simulation experience, with values above 0.8 considered good and above 0.9 considered excellent.

Statistical significance was set at *P *< 0.05 for all analyses. All statistical analyses were performed using R software (version 4.2.2; R Foundation for Statistical Computing, Vienna, Austria).

Qualitative responses were analyzed using reflexive thematic analysis following the six-step approach described by Braun and Clarke [18]. This process involved familiarization with the data, generating initial codes, searching for themes, reviewing themes, defining and naming themes, and producing the report. Two researchers independently coded the data and collaboratively identified emerging themes through an iterative process. Disagreements were resolved through discussion until a consensus was reached.

## Results

Pre-post intervention comparisons

A total of 27 medical students participated in the study (16 females, 11 males; median age 23 years, range 21-26). Most participants (74.1%) were in their fourth year of study, with the remainder in their fifth year. Participants were recruited from three UK universities. This information is summarized in Table 1.

Results summarizing the data are displayed in Tables 2-3.

**Table 2:** Pre- and post-communication skills assessment and post-session evaluation. All variables were assessed using a 5-point Likert scale (1 = lowest, 5 = highest). *P*-values are based on Wilcoxon signed-rank tests. *P < 0.05. **P < 0.001. IQR, interquartile range; CI, confidence interval

Outcome measure	Pre-session median (IQR)	Post-session median (IQR)	Median difference	95% CI for median difference	*P*-value
Perceived usefulness: How useful do you think/find ChatGPT as a simulated patient?	3 (3-5)	4 (4-5)	0.5	0.5-1	0.010*
Confidence in dealing with difficult patients	3 (2-4)	4 (4-4)	1	0.5-1.5	<0.001**
Confidence in breaking bad news	2 (2-4)	4 (4-4)	1	1-1.5	<0.001**
Confidence in counselling anxious patients	3 (2-4)	4 (4-4)	1	0.5-1.5	<0.001*

**Table 3:** Post-session feedback. All variables were assessed using a 5-point Likert scale (1 = lowest, 5 = highest). IQR, interquartile range

Post-session measure	Median (IQR)	Range
How immersive did you find the session?	4 (3-4)	1-5
I would be willing to do this again	4 (3-5)	1-5
How did you find the pace of the conversation?	3 (2-3)	2-4
How effective was ChatGPT in creating realistic scenarios?	4 (3-5)	1-5
How helpful was the feedback provided?	4 (4-5)	2-5
I would recommend ChatGPT for future sessions	4 (3-5)	1-5

**Figure 1:**
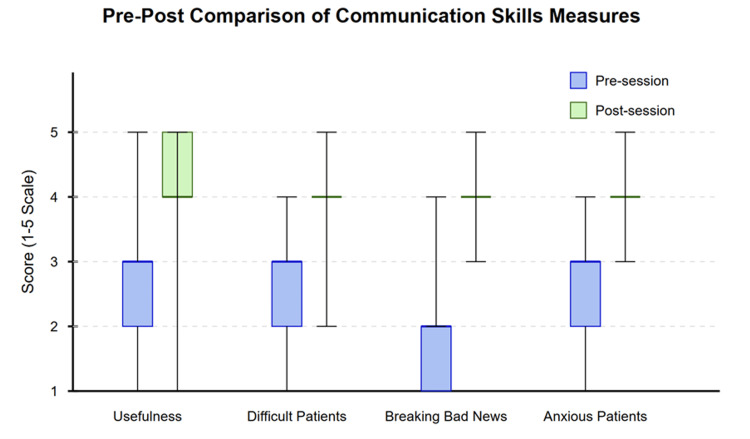
Box plot of pre-post comparison of communication skills measures with IQR. Statistical significance reflects consistent directional changes in paired measurements across participants as detected by Wilcoxon signed-rank tests. IQR, interquartile range

Wilcoxon signed-rank tests were conducted to evaluate changes in perceived usefulness of ChatGPT and confidence in communication skills before and after the simulation session (Figure 1). All measures showed statistically significant improvements.

For perceived usefulness, 20 participants reported improvement in ratings, with a significant improvement from pre- to post-session (*P *= 0.010). The median difference was 0.5 points on the 5-point scale (95% CI: 0.5-1).

All three communication skills domains showed significant improvements (all *P* < 0.001) as seen in Figure 1. For dealing with difficult patients, 18 participants reported improvement, with a median increase of 1 point (95% CI: 0.5-1.5). For breaking bad news, 20 participants reported improvement, with a median increase of 1 point (95% CI: 1-1.5). Similarly, for counselling anxious patients, 19 participants reported improvement, with a median increase of 1 point (95% CI: 0.5-1.5).

Post hoc power analysis using actual study data revealed excellent statistical power (>99.9%) for detecting changes in communication confidence measures due to large effect sizes (*d* = 1.20-1.43). For perceived usefulness (*d* = 0.51), the study achieved the conventional 80% power threshold.

University-based differences in post-session evaluations

**Figure 2:**
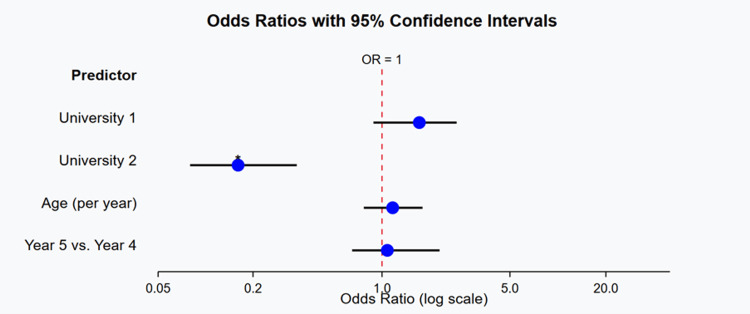
Odds ratio of regression analysis comparing demographic influences on responses.

Ordinal logistic regression analysis was conducted to examine whether university affiliation and participant characteristics influenced post-session evaluations, while controlling for age, gender, and year of study. Figure 2 represents the adjusted ORs for selected outcomes.

The regression analysis revealed that university affiliation was significantly associated with both willingness to repeat the experience and perceived effectiveness of ChatGPT in creating realistic scenarios. Specifically, comparing students from a one university had significantly lower odds of reporting higher levels of willingness to repeat the experience (OR = 0.19, 95% CI: 0.05-0.83) and lower odds of reporting higher effectiveness in creating realistic scenarios (OR = 0.18, 95% CI: 0.04-0.79). After correction for multiple comparisons, university differences in perceived realism showed a consistent pattern with university differences rating simulation aspects positively or negatively (OR = 0.13, 95% CI: 0.02-1.04, *P *= 0.12).

Age and year of study were not significantly associated with any of the post-session evaluations. The test of parallel lines confirmed that the proportional odds assumption was satisfied for both models (*P *> 0.05).

Additional post-session measures, including immersiveness, helpfulness of feedback, and recommendation for future use, showed similar patterns but did not reach statistical significance in the ordinal regression models.

Qualitative findings

Thematic analysis of open-ended responses from the post-session survey revealed four major themes regarding participants' experiences with ChatGPT as a simulated patient. Participants reflected on their initial impressions and expectations of the AI simulation, highlighted specific features they found valuable in their learning process, identified important limitations and challenges they encountered during interactions, and ultimately positioned the technology within a complementary role in medical education rather than as a standalone solution. These themes (experience and expectations, valuable features, limitations and challenges, and complementary role in education) collectively illustrate how participants perceive and interact with AI patient simulations in their developmental journey toward clinical competence.

**Figure 3:**
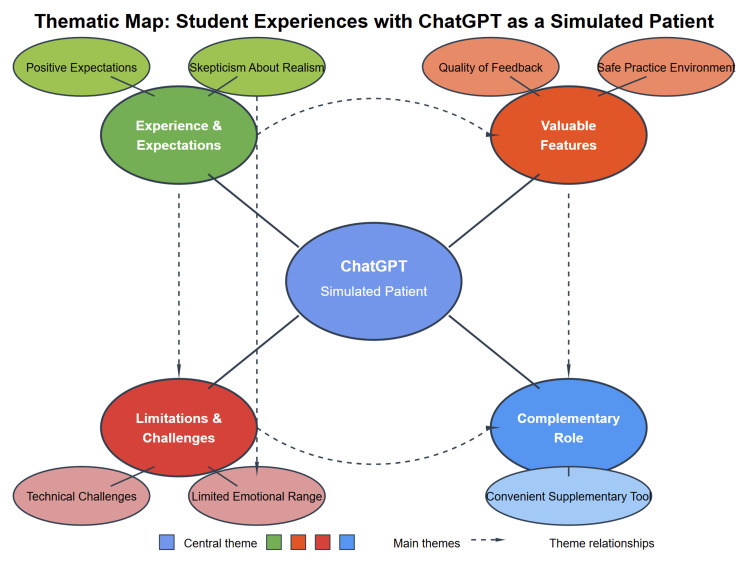
Thematic map of main theme and subthemes. Image credit: Chris Jacobs.

Figure 3 shows the thematic map and the appendices for the question list.

Experience and Expectations

Participants reported variable prior experience with AI SP. Their expectations ranged from optimistic to skeptical, with particular concern about AI's capacity to simulate authentic patient interactions.

Some participants expressed positive expectations regarding the educational potential of AI.

"High expectations - think it will be useful tool to practice." (P11)

"If it's realistic it could provide a useful tool to practice history taking at home." (P5)

Conversely, others demonstrated uncertainty about the ability of AI to accurately represent human emotions.

"I think it will be quite good but I'm unsure of how it will be able to convey emotions." (P17)

"It will be unrealistic." (P6)

Valuable Features

Participants identified specific aspects of the AI simulation that enhanced their learning experience. The quality of feedback and the creation of a psychologically safe practice environment emerged as particularly beneficial elements.

The detailed, personalized feedback provided by ChatGPT was generally noted as a strength.

"Personalised feedback from chatGPT - it was very specific, and had a good mix of positive points and things to do better next time." (P2)

"Feedback was really detailed and helpful - helped me to think about my language and phrases to use." (P10)

Participants also valued the low-pressure environment for practicing challenging communication scenarios.

"Felt less pressure than talking to a real person as you know it doesn't have emotions." (P13)

"It was useful to practice having difficult conversations as this is not something you get a lot of practice of as a medical student." (P12)

Limitations and Challenges

Technical issues and limitations in emotional expression were identified as primary challenges when using ChatGPT as an SP.

Technical difficulties affected some participants' experience.

"Slight glitch as chatGPT did not work for the second scenario, and only gave feedback in text form rather than speaking aloud." (P2)

Participants also noted limitations in the AI’s ability to convey emotional nuance.

"I think the limitation was chat GPT's emotional range. This will come with updates of the software!" (P17)

Complementary Role in Education

Participants positioned AI as a valuable complement to existing educational approaches rather than a replacement for traditional methods. The convenience and accessibility of AI-based practice were particularly valued.

Participants described ChatGPT as a useful supplementary tool.

"… the tool is really useful, obviously not as useful as an actor but this is very difficult to arrange and this is a really convenient and easy way to practice." (P27)

"That it might be useful for us to use in our own time - I probably wouldn't like to have my university replace this with actual actors." (P18)

These findings suggest that while AI SP have limitations, they offer distinct advantages in terms of accessibility and convenience that could complement traditional standardized patient encounters in medical education.

Field notes on LLM behavior

Observational field notes revealed consistent patterns in AI feedback structure and interaction dynamics. The LLM consistently provided three-part feedback: positive observations, improvement suggestions, and overall impression. However, exclusively from a patient perspective rather than evaluating clinical accuracy. Notable feedback included suggestions for translating medical terminology, for example, when a student used "missed miscarriage," ChatGPT suggested saying "It is when the baby has sadly stopped growing, but the body has not yet recognized the loss." The AI consistently recommended more empathetic phrasing, such as replacing "it is best to manage this conservatively" with "We will take a gentle approach..." It also identified potentially distressing language, recommending "tissues from the pregnancy might pass naturally over the coming days" instead of a student's phrase "bits of your pregnancy left." Interaction limitations were observed when students exhibited natural communication patterns involving pauses or hesitations; in these instances, ChatGPT frequently interrupted, prematurely concluded scenarios, or inappropriately shifted into the physician role despite explicit instructions to the contrary in the system prompt.

## Discussion

This mixed-methods study evaluating ChatGPT as an SP for communication skills training demonstrated significant improvements in medical students' self-reported confidence across all measured domains. Following the ChatGPT simulation, students reported substantial increases in confidence for dealing with difficult patients, breaking bad news, and counseling anxious patients (all *P *< 0.001). Perceived usefulness of AI simulation also increased significantly after exposure (*P *= 0.010). Qualitative analysis revealed four key themes: varying expectations, valuable feedback features, technical and emotional limitations, and AI's complementary role in education. University affiliation appeared to influence perceptions, with students from one university consistently rating the realism of the simulation lower (OR = 0.13, 95% CI: 0.02-1.04).

These findings align with previous research showing that simulation-based learning can enhance communication self-efficacy [2,6]. The median increase of one point across all domains represents a meaningful improvement on a Likert scale [19], suggesting that even brief exposure to AI SPs can boost confidence in challenging communication scenarios. The ordinal logistic regression revealed differences between universities, with groups of students reporting different willingness to repeat the experience and lower ratings of ChatGPT's effectiveness. These institutional differences were not explained by age or year of study, suggesting that contextual factors might exist, such as prior exposure to simulation, curricular emphasis on communication skills, or institutional culture, may influence receptiveness to AI in general. This study highlights that it can be difficult to work out the effects of an educational intervention across institutions. A similar finding was made in a study reviewing case-based learning in healthcare education [20].

The observed differences in perception across universities may reflect institutional cultures that influence technology acceptance and implementation. Additionally, these variations might stem from differences in curriculum integration of technology, institutional messaging around AI, or previous exposure to simulation-based education. Medical schools with established simulation programs may foster environments where students develop expectations and evaluation frameworks that align with technological innovations. Alternatively, institutions with strong traditions of actor-based simulation might cultivate preferences for human interaction that influence technology perception. The Technology Acceptance Model suggests that perceived usefulness and ease of use are mediated by institutional and social factors that shape individual attitudes.

Our qualitative findings provided further insights into how AI-supported learning works. Participants valued the detailed and personalized feedback that ChatGPT provided, which is consistent with established principles of effective feedback in medical education [21]. The safe practice environment created by AI simulation, where students could practice without fear of causing distress to real or SPs, had emerged as particularly valuable. Psychological safety of conversational AI has been reported in previous studies. The qualitative themes of "Limitations and Challenges" and "Complementary Role in Education" provide important context for implementing AI SPs. Participants identified technological issues and limitations in emotional range as key challenges, yet still considered AI as a valuable supplement rather than a replacement for traditional methods. This aligns with characterization of the information age, in that, medical education must integrate AI thoughtfully rather than uncritically embracing or rejecting it [22]. Also, field notes observations highlight both the strengths of LLM for certain feedback in communication skills training and the current technological limitations in processing natural conversational rhythms.

The findings extend previous research on VP in several ways. Unlike earlier text-based VP [23], advanced LLM with speech provides dynamic, conversational interactions that more closely approximate real-time clinical communication. Participants in our study specifically valued this responsive nature. The improvements in self-reported confidence are similar to findings that virtual patient simulations can effectively develop communication skills [24]. However, our qualitative data highlight limitations in emotional authenticity that have not been prominently discussed in previous literature. As AI technologies continue to develop, addressing these emotional limitations will be crucial for creating more authentic simulated encounters. Simulation-based training in other qualitative exploration improved medical students' communication skills in various scenarios [25]. Our study builds on this by exploring how AI-driven simulation might offer a more scalable alternative to traditional actor-based simulation approaches while maintaining similar benefits for learner confidence and psychological safety.

Our findings suggest several practical applications for medical educators. AI-based SPs could serve as an accessible supplement to traditional methods, particularly for independent practice. This may be especially valuable given the logistical challenges and costs associated with SP programs [4].

Limitations

Several limitations are to be acknowledged. Our assessment relied on self-reported confidence rather than objective measures of communication competence. While self-confidence is important, it may not directly translate to improved clinical performance [26]. While our post hoc power analysis indicated the study was adequately powered to detect the observed large effects on communication confidence (*d *= 1.20-1.43, power > 99.9%), the relatively small sample size (*N *= 27) limited our ability to detect smaller effects and perform more sophisticated subgroup analyses, particularly regarding university differences. The unequal distribution of participants across universities further constrained these comparisons. Finally, the study evaluated only immediate post-session perceptions, longer-term follow-up would be valuable to assess retention of confidence. 

Future research

Further research should examine the impact of AI SP encounters on objective measures of communication competence, ideally through observed structured clinical examinations or actual patient interactions. Longitudinal studies could assess whether improvements in confidence persist over time and translate into clinical practice. With advancing LLM, generative AI achieves increasingly realistic interactions, warranting further investigation into its applications and impacts.

## Conclusions

This mixed-methods exploratory study provides preliminary evidence that ChatGPT conversational AI may serve as a supplementary tool for practicing challenging communication skills, with participants demonstrating significant improvements in self-reported confidence across multiple domains. While our findings are promising, they should be interpreted within the methodological constraints of our pre-post design, modest sample size, and reliance on subjective measures, factors that limit definitive conclusions about efficacy. Our qualitative analysis identified both strengths (detailed feedback, psychological safety, accessibility) and limitations (emotional authenticity constraints, technical challenges, institutional variation in acceptance) of the AI approach. Rather than proposing replacement of established methods, our exploratory findings suggest that AI systems might complement traditional standardized patient encounters by expanding practice opportunities beyond scheduled sessions. Future research employing controlled designs, objective skill assessments, and investigation of skill transfer to clinical settings is needed to establish the true educational value of conversational AI in communication skills training.

## Appendices

Appendix

Scenario 1: Angry patient: Mr Riccardo is 25 years old and has been recently diagnosed with Chlamydia. You are a FY1 in a Sexual Health clinic. Please take a short history and talk to the patient about partner notification.

Prompt 1 (Arbor voice): ChatGPT can you please pretend to be a patient while a medical student acting as an FY1 doctor in the NHS takes history and converses with you? Do not take on a doctor role and don’t narrate your expressions or actions at any point.

You are Mr Riccardo (25-year-old male) who has been recently diagnosed with Chlamydia, but you are angry and do not want to inform your partner. You are in the sexual Health clinic.

Please allow for the student to take time to pause and think of questions. Do not stop pretending to be a patient until we say the end of conversation.

Scenario 2: Breaking bad news: Mrs. Brown is a 23-year-old lady. G1P0, who is 8 weeks pregnant and has been diagnosed with a missed miscarriage by ultrasound scan.

You are a FY1 in Early Pregnancy unit, please communicate the scan result to the patient.

Prompt 2 (Sol voice): ChatGPT can you please pretend to be a patient while a medical student acting as an FY1 doctor in the NHS takes history and converses with you? Do not take on a doctor role and don’t narrate your expressions or actions at any point.

The scenario is that you are Mrs. Brown, a 23-year-old lady. G1P0, who is 8 weeks pregnant and have come into the Early Pregnancy Unit today following some bleeding. You have had an ultrasound scan this morning in the EPU and you are waiting for results. You will eventually be diagnosed with miscarriage, and you will be very upset and teary.

Please allow for the student to take time to pause and think of questions. Do not stop pretending to be a patient until we say the end of conversation.

Scenario 3: Mrs Patel is 27-year-old, G1P0, 32/40 presenting to antenatal clinic after experiencing a sudden gush of fluid earlier today and has been diagnosed with spontaneous premature rupture of membrane. You are an FY1 doctor in an antenatal clinic. Please counsel the patient about her diagnosis and next steps.

Prompt 3 (Sol voice): ChatGPT can you please pretend to be a patient while a medical student acting as an FY1 doctor in the NHS takes history and converses with you? Do not take on a doctor role and don’t narrate your expressions or actions at any point.

You are Mrs Patel is 27-year-old, G1P0, 32/40 presenting to antenatal clinic after being diagnosed with Premature spontaneous rupture of membrane. You are very anxious and worried about your baby and need counselling about further steps.

Please allow for the student to take time to pause and think of questions. Do not stop pretending to be a patient until we say the end of conversation.

Communication skills role-play using ChatGPT (Pre session)  Thank you for your participation!  * Required 1. 2. 3. 4. 5. Date of your session * Please state your age * Enter your answer Which university and year group do you belong to? * Bristol Year 4 Oxford Year 5 King's Year 4 Please state your gender * Female Male Non-binary Prefer not to say Do you know that there are AI models that can simulate patients? * Yes No Communication skills role-play using ChatGPT (Pre-session)  02/04/2025, 10:52 6. 7. 8. 9. How confident do you feel in the following communication skills? * Extremely confident Dealing with difficult patients Breaking bad news Counselling anxious patients Somewhat confident Neutral Not so confident How useful do you think ChatGPT as a simulated patient would be for practicing communication skills? * Very useful Not very useful Extremely not confident I'm not sure Have you had prior experience practicing difficult communication with simulated patients? If yes, please briefly describe. * Enter your answer What expectations do you have for using AI as a tool in this training? *

Communication skills role-play using ChatGPT (Post session) Thank you for your participation!  * Required 1. 2. 3. 4. 5. Date of your session * Please state your age * Enter your answer Which university and year group do you belong to? * Bristol Year 4 Oxford Year 5 King's Year 4 Please state your gender * Female Male Non-binary Prefer not to say How confident do you feel after this session in the following communication skills? * Extremely confident Dealing with difficult patients Breaking bad news Counselling anxious patients Slightly confident Neutral Not so confident Extremely not confident Page 1 of 3 How immersive did you find the session (e.g., did it feel like a real-life patient encounter)? * 6. Very immersive Somewhat immersive Neutral Not so immersive Not immersive at all I would be willing to do this again because it has value to me. * 7. Strongly agree Agree Neither agree nor disagree Disagree Strongly disagree How did you find the pace of the conversation with ChatGPT? * 8. Very rushed Somewhat rushed Not too rushed or slow Somewhat slow Very slow How useful did you find ChatGPT as a simulated patient for practicing communication skills? * 9. Very useful Somewhat useful Neutral Somewhat not useful Not useful at all How effective was ChatGPT as simulated patients in creating a realistic scenarios? * 10. Very effective Somewhat effective Neutral Not so effective Not very effective How helpful was the feedback provided by ChatGPT as the patient during the session? * 11. Very helpful Somewhat helpful Neither helpful nor unhelpful Somewhat unhelpful Very unhelpful Communication skills role-play using ChatGPT (Post-session) 02/04/2025, 10:53 12. 13. 14. 15. I would recommend using ChatGPT as a simulated patients for future session. * Strongly agree Agree Neither agree nor disagree Disagree Strongly disagree Did the absence of a physical patient affect your sense of realism or engagement during the session? If so, please describe how. * Enter your answer What aspects of the session did you find most helpful for your learning? * Enter your answer Do you have any additional suggestions for improving the overall quality of this session? *
